# Spatial, temporal, and space-time clusters associated with opioid and cannabis poisoning events in U.S. dogs (2005–2014)

**DOI:** 10.1371/journal.pone.0266883

**Published:** 2022-04-28

**Authors:** Mohammad Howard-Azzeh, David L. Pearl, Olaf Berke, Terri L. O’Sullivan

**Affiliations:** Department of Population Medicine, University of Guelph, Guelph, Ontario, Canada; University of Lincoln, UNITED KINGDOM

## Abstract

While a substantial amount of research has focused on the abuse of opioids and cannabinoids in human populations, few studies have investigated accidental poisoning events in pet populations. The objective of this study was to identify whether poisoning events involving opioids and cannabinoids clustered in space, time, and space-time, and compare the locations of clusters between the two toxicants. Data were obtained concerning reports of dog poisoning events from the American Society for the Prevention of Cruelty to Animals’ (ASPCA) Animal Poisoning Control Center (APCC), from 2005–2014. The spatial scan statistic was used to identify clusters with a high proportion of these poisoning events. Our analyses show that opioid and cannabinoid poisoning events clustered in space, time, and space-time. The cluster patterns identified for each toxicant were distinct, but both shared some similarities with human use data. This study may help increase awareness to the public, public health, and veterinary communities about where and when dogs were most affected by opioid and cannabinoid poisonings. This study highlights the need to educate dog owners about safeguarding opioid and cannabinoid products from vulnerable populations.

## Introduction

Opioids and cannabinoids are amongst the most widely used drugs by humans in the USA [1]. With the continued increase in opioid related deaths, along with the rapid increased use of cannabinoids and relaxed cannabis legalisation [1–5], understanding the impact of the use of these drugs on vulnerable populations is of growing importance.

Several studies have found associations between adult opioid use and accidental opioid poisonings in pediatric populations [6–9]. Similarly, research shows there may be an association between cannabis legalisation and an increase in unintentional poisoning events in children [10]. Like children, dogs are vulnerable to the effects of human drug use. Yet, research focused on the impact of adult opioid and cannabinoid use on dog populations has only recently received attention [11–15]. These studies identified risk factors at the community and dog levels, such as legislation, prescription rate, urbanicity, income disparity, time, sex, weight, age, and reproductive status, that were associated with accidental dog poisonings [12, 13]. However, little is known about the distribution and frequency of these dog poisoning events in space and time.

Studies indicate the general distribution of opioid and cannabinoid use in humans to be substantially different in space and time. Research suggests that humans in rural communities suffer disproportionately from opioid misuse compared to those in urban communities [16, 17], though there seems to be considerable variation in opioid abuse between different rural environments [18]. Whereas cannabinoids appear to be the opposite, being used proportionally more in urban than rural environments [19, 20], which appears to be consistent with cannabinoid poisonings in dogs [13]. The frequency of US dog opioid poisonings appeared to increase from 2006 to 2008 but decreased from 2009 to its lowest in 2014, whereas the frequency of dog cannabinoid poisonings appeared to increase from 2009 to 2014 [12, 13, 15].

Based on 2017 data at the county-level, it appeared that human opioid abuse was concentrated in the mideastern US surrounding Appalachia [21, 22]. At the state and substate-levels, proportional human cannabinoid use tended to group into four regions: northwest region around Washington, Oregon, and northern California; the far northeast around Maine, Vermont, New Hampshire, Massachusetts, Rhode Island, and Connecticut; in Colorado; and the District of Columbia [23, 24]. It would be important to understand if dogs in these regions were at an elevated risk of opioid or cannabinoid poisonings to help understand opioid and cannabinoid poisonings beyond a simple rural/urban comparison.

To date, no published studies have focused on identifying regions or periods where/when dogs are at a particularly high risk of either an opioid or cannabinoid poisoning event. This information would help direct veterinarians and public health to be better prepared for opioid or cannabinoid poisonings, and possibly guide future research and interventions in high-risk regions. Therefore, the objectives of this study were to identify clusters of high risk in space, time, and space-time for opioid and cannabinoid poisoning events in US dogs using data from a national animal poison control center, and assess how well they correspond to known human areas of high abuse of these substances.

## Methods

### Data

The data used in this study were collected by the American Society for the Prevention of Cruelty to Animals (ASPCA) through their Animal Poison Control Center (APCC) services. The APCC provides over-the-phone emergency toxicology advice to the public, veterinarians, and other poison control centers needing assistance dealing with a potentially poisoned animal. The services for each case cost 65 USD and is not a mandatory service for any party. With each call, the APCC collects data concerning patient characteristics, toxicant information, clinical effects, outcome, and location/date/time of the call. These data are stored in the APCC’s animal toxicology database (AnTox). This study uses APCC’s AnTox data from 2005–2014 and only explores data from the continental U.S. (i.e., does not include calls from Alaska, Hawaii, or U.S. territories).

A call to the APCC reporting more than one potentially poisoned animal from the same household was considered a single unique observation. Since our analyses were performed at the household level, the data used in these analyses included 206,266 household calls. For each observation in this study, information regarding toxicant exposure and location/date/time were obtained from the AnTox database.

For the analyses concerning opioid poisonings, an opioid call was defined as any call to the APCC from a household that involved at least one dog exposed to an opioid. This included all prescription and illicit opioids as well as over the counter drugs containing opioids that could be abused. A call that presented a dog with multiple toxicant exposures was still considered an opioid exposure if one of those toxicants was an opioid. Non-opioid calls included any call to the APCC from a household involving dog(s) that were only exposed to non-opioid toxicants.

A cannabis call was defined as any call to the APCC from a household involving at least one dog that was exposed to any form of cannabis or cannabis derivative, including synthetic cannabinoids. Cannabis products were often present in edible products, such as brownies. If a dog was exposed to a cannabis product and another toxicant at the time of the call to the APCC, it was also considered a cannabis call. A non-cannabis call was defined as a call to the APCC from a household involving dog(s) only exposed to non-cannabis related toxicants. If a call concerned a dog that was exposed to both an opioid and a cannabinoid, it was considered a positive exposure for the respective analyses concerning each toxicant (n = 32).

### Spatial and temporal analyses

Scan statistics were used to identify clusters of opioid and cannabinoid cases in space, time, and space-time [25]. Bernoulli models were used to identify clusters in space/time/space-time, analysing the ratio of either opioid calls to non-opioid calls or cannabis calls to non-cannabis calls. Additionally, space-time clusters were also identified with space-time permutation models using only opioid calls or cannabinoid calls. The space-time permutation models correct for purely spatial or temporal clusters that may bias space-time analyses [26].

No maximum value was set for the scanning window’s radius as human populations are not evenly distributed. A maximum cluster spatial scanning window of 50% of the study population was used since there was no *a priori* hypothesis concerning the maximum size of these clusters. This allows for the identification of both small and large clusters and minimizes pre-selection bias [25]. Each scan was done in one-year increments (e.g., January 1, 2005 to December 31, 2005) and using the entire study period (i.e., January 1, 2005 to December 31, 2014), with a maximum temporal window of 50% of the study period (i.e., 6 months for annual scans or 5 years for scans of the entire study period). Due to issues with computational speed, temporal precision was set to one month for space-time analyses, but to the day for temporal analyses. Standard Monte Carlo estimations (999 replications) were used for the purely temporal and spatial analyses. For space-time Bernoulli and space-time permutation models, sequential Monte Carlo estimations (999 replications) with early termination cut-offs of 50 replications (which terminates Monte Carlo simulations at 50 replications when p-values are large) were used to deal with excessive computational times. For spatial and temporal analyses, only clusters without spatial or temporal overlap were reported, respectively. For space-time analyses, all clusters with no pairs of centres within each other’s clusters were allowed to identify space-time clusters that overlapped in space, but not time. However, only the most likely non-overlapping clusters in space-time were reported. For each cluster, the p-value, relative risk for Bernoulli models, observed to expected ratio, radius, and the location in space/time/space-time were reported. All scan statistics were performed using SaTScan v. 9.6 with one-tailed hypotheses (α = 0.05) to identify clusters of high levels of opioid or cannabinoid related calls [25]. Statistically significant spatial and space-time clusters were located and visualised on maps using QGIS v.3.0.2. The coordinate reference system used for all map projections in this study was the World Geodetic System 84 from the European Petroleum Survey Group Parameter Dataset (EPSG:4326—WGS 84).

Choropleth maps depicting the proportion of opioid calls compared to all toxicant calls and cannabinoid calls compared to all toxicant calls by state were created and mapped using QGIS v.3.0.2. Choropleth maps were made using data from the entire study period.

With regards to discussions concerning the use of the phrase "statistically significant" [27], in this manuscript, the term "statistically significant" is not intended to infer biological/epidemiological importance, or causation. It is used to indicate that based on our statistical criteria, we have enough evidence to infer that the measure of association for a given predictor variable or contrast is different from the null value [12]. The term “statistical significance” was used to describe results in an exploratory sense since our study involved a pre-existing dataset [28].

## Results

Opioid and cannabinoid poisoning calls accounted for 2.77% and 0.99% of all poisoning calls, respectively (Table 1). Opioid calls were at their highest in 2008 (3.26%) and their lowest in 2014 (2.19%) (Table 1). Cannabinoid calls generally increased throughout the study where they were at their lowest in 2006 (0.65%) and at their highest in 2014 (1.53%) (Table 1).

**Table 1:** Proportion of household-level US dog-associated poisoning calls to the APCC^a^ related to an opioid or cannabinoid for each year of the study (2005–2014).

Year	Opioid Calls	Cannabinoid Calls	Total
**2005**	474 (3.21%)	101 (0.68%)	14767
**2006**	512 (2.93%)	114 (0.65%)	17490
**2007**	624 (3.09%)	180 (0.89%)	20184
**2008**	697 (3.26%)	170 (0.80%)	21359
**2009**	668 (3.15%)	179 (0.84%)	21199
**2010**	623 (2.89%)	226 (1.05%)	21584
**2011**	580 (2.67%)	254 (1.17%)	21750
**2012**	520 (2.38%)	217 (1.00%)	21807
**2013**	510 (2.25%)	242 (1.07%)	22711
**2014**	512 (2.19%)	359 (1.53%)	23415
**Total**	5720 (2.77%)	2042 (0.99%)	206266

**Fig 1:**
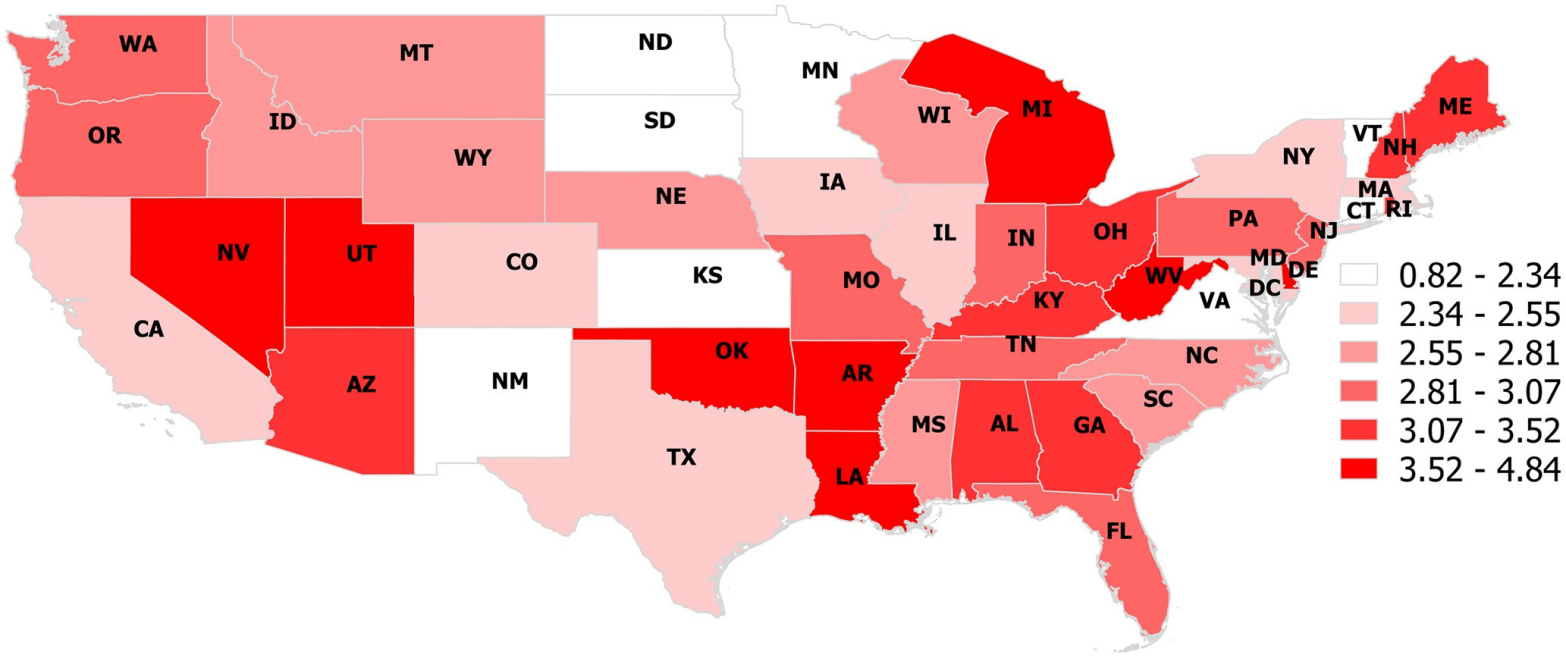
Choropleth map illustrating the proportion of dog-associated poisoning calls to the Animal Poison Control Center that were related to an opioid poisoning in each continental US state (2005–2014).

**Fig 2:**
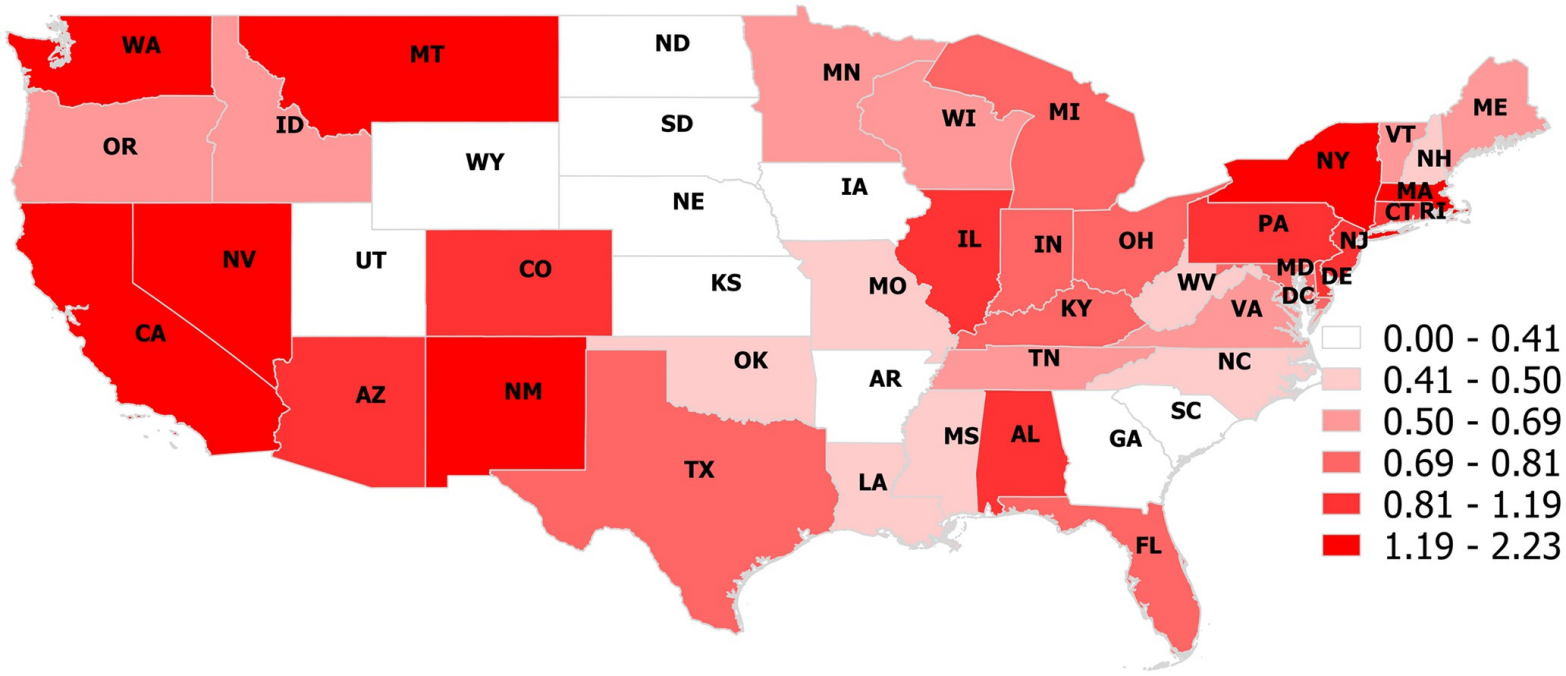
Choropleth map illustrating the proportion of dog-associated poisoning calls to the Animal Poison Control Center that were related to a cannabinoid poisoning in each continental US state (2005–2014).

The choropleth maps depict the proportion of opioid (Fig 1) and cannabinoid (Fig 2) poisoning calls for each state the study period. The proportion of calls from households concerning opioids ranged from 0.82% in the District of Columbia to 4.84% in West Virginia (Fig 1 and Table 2). The proportion of calls from households concerning cannabinoids ranged from 0% in Wyoming, South Dakota, Nebraska, and North Dakota, to 2.23% in Montana (Fig 2 & Table 2).

**Table 2:** Proportion of all household dog-associated poisoning calls to the APCC^a^ related to an opioid or cannabinoid in each continental US state (2005–2014).

State	Opioid calls	Cannabis calls	Total Calls
**Alabama (AL)**	14 (3.22%)	4 (0.92%)	435
**Arkansas (AR)**	14 (4.02%)	1 (0.29%)	348
**Arizona (AZ)**	191 (3.34%)	57 (1.00%)	5714
**California (CA)**	633 (2.55%)	386 (1.56%)	24792
**Colorado (CO)**	111 (2.50%)	45 (1.01%)	4434
**Connecticut (CT)**	175 (2.14%)	89 (1.09%)	8175
**District of Columbia (DC)**	7 (0.82%)	15 (1.77%)	849
**Delaware (DE)**	40 (3.66%)	13 (1.19%)	1094
**Florida (FL)**	312 (3.06%)	77 (0.75%)	10207
**Georgia (GA)**	91 (3.09%)	12 (0.41%)	2942
**Iowa (IA)**	18 (2.44%)	3 (0.41%)	737
**Idaho (ID)**	15 (2.81%)	3 (0.56%)	534
**Illinois (IL)**	290 (2.51%)	95 (0.82%)	11544
**Indiana (IN)**	92 (3.07%)	24 (0.80%)	2993
**Kansas (KS)**	17 (2.34%)	1 (0.14%)	728
**Kentucky (KY)**	43 (3.52%)	9 (0.74%)	1222
**Louisiana (LA)**	50 (4.38%)	5 (0.44%)	1141
**Massachusetts (MA)**	264 (2.49%)	130 (1.23%)	10611
**Maryland (MD)**	161 (2.46%)	50 (0.77%)	6533
**Maine (ME)**	36 (3.38%)	6 (0.56%)	1064
**Michigan (MI)**	208 (4.02%)	42 (0.81%)	5175
**Minnesota (MN)**	41 (2.02%)	14 (0.69%)	2029
**Missouri (MO)**	41 (3.04%)	6 (0.44%)	1350
**Mississippi (MS)**	6 (2.76%)	1 (0.46%)	217
**Montana (MT)**	7 (2.60%)	6 (2.23%)	269
**North Carolina (NC)**	176 (2.67%)	33 (0.50%)	6588
**North Dakota (ND)**	1 (1.33%)	0 (0.00%)	75
**Nebraska (NE)**	7 (2.72%)	0 (0.00%)	257
**New Hampshire (NH)**	45 (3.42%)	6 (0.46%)	1316
**New Jersey (NJ)**	388 (3.06%)	121 (0.96%)	12663
**New Mexico (NM)**	20 (2.22%)	11 (1.22%)	902
**Nevada (NV)**	102 (3.69%)	35 (1.27%)	2763
**New York (NY)**	397 (2.42%)	297 (1.81%)	16431
**Ohio (OH)**	253 (3.43%)	56 (0.76%)	7380
**Oklahoma (OK)**	19 (4.53%)	2 (0.48%)	419
**Oregon (OR)**	86 (3.05%)	19 (0.67%)	2820
**Pennsylvania (PA)**	374 (3.06%)	105 (0.86%)	12203
**Rhode Island (RI)**	42 (3.17%)	10 (0.76%)	1323
**South Carolina (SC)**	60 (2.58%)	9 (0.39%)	2326
**South Dakota (SD)**	1 (1.03%)	0 (0.00%)	97
**Tennessee (TN)**	64 (3.06%)	13 (0.62%)	2090
**Texas (TX)**	297 (2.50%)	83 (0.70%)	11864
**Utah (UT)**	22 (4.48%)	1 (0.20%)	491
**Virginia (VA)**	224 (2.16%)	63 (0.61%)	10376
**Vermont (VT)**	9 (2.02%)	3 (0.67%)	445
**Washington (WA)**	110 (3.03%)	53 (1.46%)	3629
**Wisconsin (WI)**	104 (2.72%)	25 (0.65%)	3821
**West Virginia (WV)**	34 (4.84%)	3 (0.43%)	702
**Wyoming (WY)**	4 (2.80%)	0 (0.00%)	143
**Total**	5716(2.77%)	2042 (0.99%)	206261

^a^Animal Poison Control Center.

### Opioid analyses

#### Purely spatial clusters

**Fig 3:**
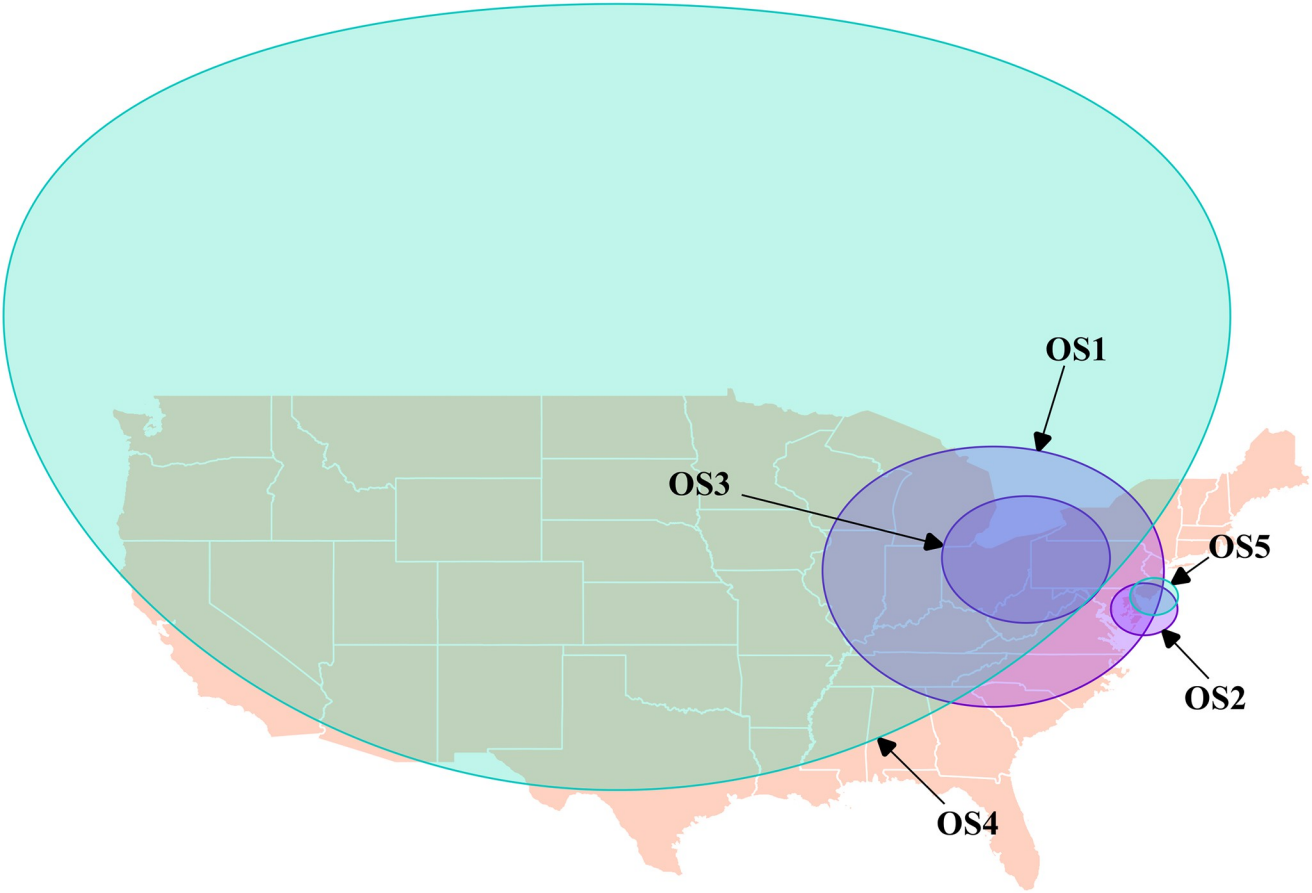
Locations of statistically significant spatial clusters identified with the spatial scan statistic using Bernoulli models, where there were statistically significant higher proportions of dog-associated poisoning calls to the Animal Poison Control Center related to opioids in the continental US (2005–2014).

The purely spatial scan identified 5 statistically significant spatial clusters of proportionally high dog opioid poisoning calls (Fig 3 & Table 3). Four of these clusters were identified in the northeastern region of the country. Cluster OS1 (Opioid Spatial 1) was identified in a scan of the 2005 data, and it covered most of the northeastern interior, and was centered just east of Columbus, Ohio (Fig 3 & Table 3). In a scan of 2007 data, cluster OS2 covered Delaware, southern New Jersey, and eastern Maryland, and centered over Rohoth, Delaware (Fig 3 & Table 3). Cluster OS3 was identified when we scanned the 2010 data, and was centered over Youngstown, Ohio (Fig 3 & Table 3). When the spatial analysis was performed with data from the entire study period, 2 spatial clusters were identified. OS4 was a large cluster covering most of the US’s interior, and was centered north of Bottineau, North Dakota (Fig 3 & Table 3). OS5’s location was similar to OS2, centered over Somers Point, New Jersey, and covered most of Delaware and southern New Jersey (Fig 3 & Table 3).

**Table 3:** Purely spatial clusters identified with the spatial scan statistic using Bernoulli models, where there were statistically significant higher proportions of dog-associated poisoning calls to the APCC^a^ related to opioids in the continental US (2005–2014).

Cluster	Year(s) Scanned	O/E^b^	Relative Risk	Observed	Expected	P-Value	Latitude	Longitude	Radius (Km)
OS1	2005	**1.27**	**1.57**	250	197.15	0.028	40.27	-82.33	694.72
OS2	2007	**2.24**	**2.34**	44	19.60	0.035	38.71	-75.08	139.03
OS3	2010	**1.89**	**2.01**	79	41.88	0.004	41.10	-80.76	338.23
OS4	2005–2014	**1.12**	**1.22**	2512	2240.90	<0.001	48.91	-100.42	2097.84
OS5	2005–2014	**1.47**	**1.49**	265	180.53	<0.001	39.32	-74.60	99.20

^a^Animal Poison Control Center

^b^Observed/Expected

#### Purely temporal clusters

When the scan test was performed to identify purely temporal clusters of proportionally high opioid calls, no clusters were identified by year (Table 4). When the scan tests were performed for the entire study period, cluster OT1 (Opioid Temporal 1) was identified, lasting from May 12, 2005 to March 28, 2010 (Table 4).

**Table 4:** Purely temporal clusters identified with the spatial scan statistic using Bernoulli models, where there were statistically significant high proportions of dog-associated poisoning calls to APCC^a^ related to opioids or cannabinoids in the continental US (2005–2014).

Cluster	Toxin	Year(s) Scanned	Start Date	End Date	O/E^b^	Relative Risk	Observed	Expected	P-Value
OT1*	Opioid	2005	5/12/2005	3/28/2010	**1.13**	**1.28**	2981	2631.30	0.001
CT1*	Cannabinoid	2005–2014	6/29/2010	12/28/2014	**1.20**	**1.50**	1200	995.87	0.001

^a^Animal Poison Control Center

^b^Observed/Expected

*No temporal clusters were identified when the analyses were performed by year

#### Space-Time clusters

**Fig 4:**
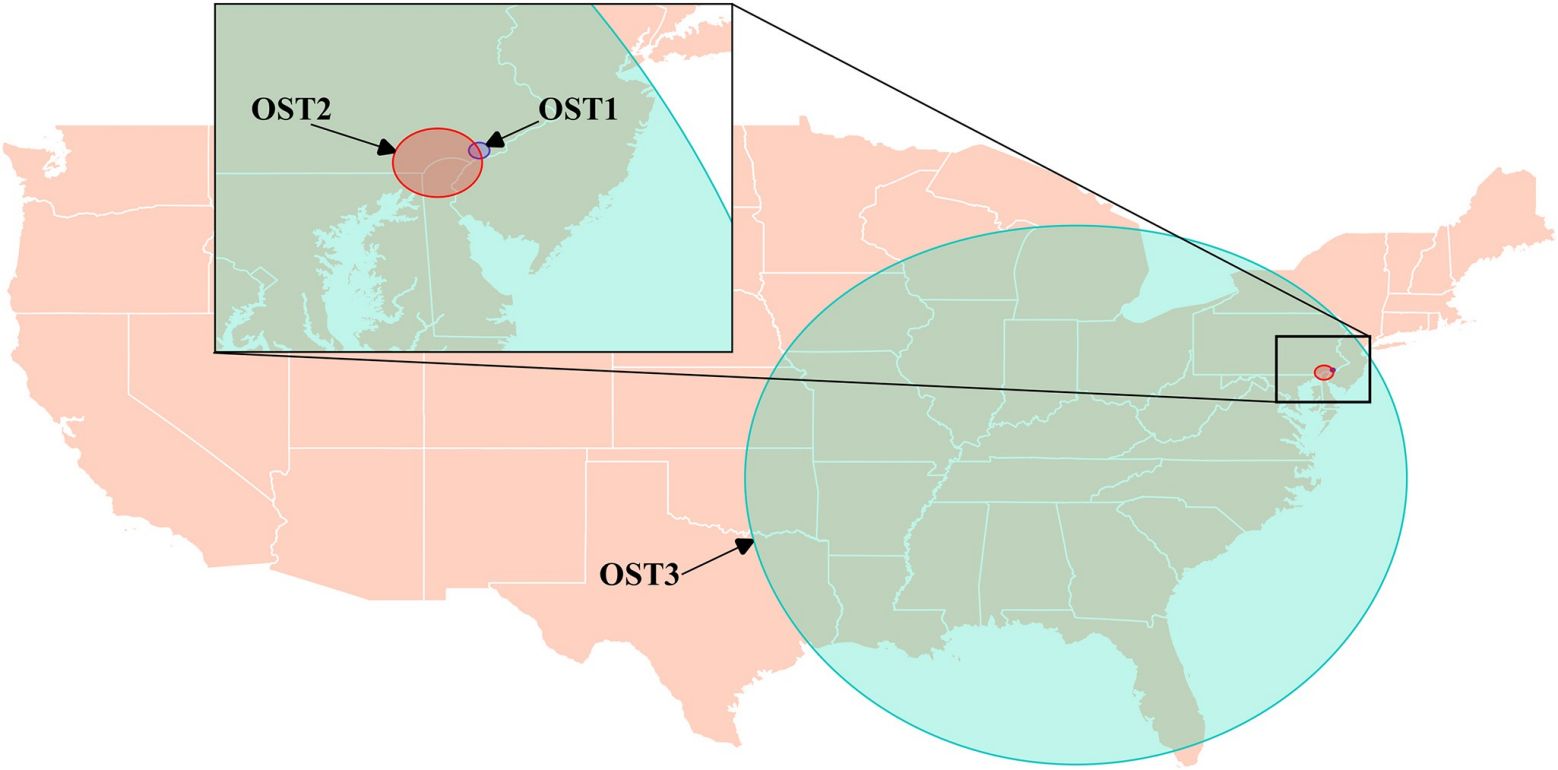
Locations of statistically significant space-time clusters of high levels of calls concerning opioids identified using the spatial scan statistic with Bernoulli and space-time permutation models, based on data from dog-associated poisoning calls to the Animal Poison Control Center related to opioids in the continental US (2005–2014).

The statistically significant space-time clusters, based on scan tests of individual years, identified were in the same region as the purely spatial clusters OS2 and OS5, the area where Delaware, Pennsylvania, Maryland, and New Jersey meet, as well as most of the east coast of the US. The space-time Bernoulli model and the space-time permutation model both identified 1 space-time cluster of proportionally high opioid calls (Fig 4 & Table 5). The space-time Bernoulli model identified cluster OST1 (Opioid Space-Time 1), which covered southwest Philadelphia in the center of Delaware county, Pennsylvania, and occurred from February 1 to May 31, 2007 (Fig 4 & Table 5). The space-time permutation model identified cluster OST2, which covered northern Delaware, southern Pennsylvania, eastern Maryland, and parts of southwestern New Jersey, and occurred from September 1 to September 30, 2008 (Fig 4 & Table 5). When the analysis was performed on the entire study period using the space-time Bernoulli model, we identified a large cluster that covered most of the eastern half of USA, centered north of Cleveland, Tennessee, and occurred from June 1, 2005 to March 31, 2010 (Fig 4 & Table 5). No clusters were identified when the space-time permutation model was applied to the entire study period.

**Table 5:** Space-time clusters identified with the spatial scan statistic using Bernoulli and space-time permutation models, where there were statistically significant higher levels of dog-associated poisoning calls to the APCC^a^ related to opioids in the continental US (2005–2014).

Cluster	Model Type	Year(s) Scanned	Start Date	End Date	O/E^b^	Relative Risk	Observed	Expected	P-Value	Latitude	Longitude	Radius (Km)
OST1	Space-Time Bernoulli	2007	2/1/2007	5/31/2007	**18.87**	**19.07**	7	0.37	0.017	39.90	-75.35	6.88
OST2	Space-Time Permutation	2008	9/1/2008	9/30/2008	**9.14**	N/A	8	0.88	0.012	39.80	-75.67	29.55
OST3	Space-Time Bernoulli	2005–2014	6/1/2005	3/31/2010	**1.23**	**1.32**	1620	1319.81	0.001	35.26	-84.87	1110.60

^a^Animal Poison Control Center

^b^Observed/Expected

### Cannabis

#### Purely spatial clusters

**Fig 5:**
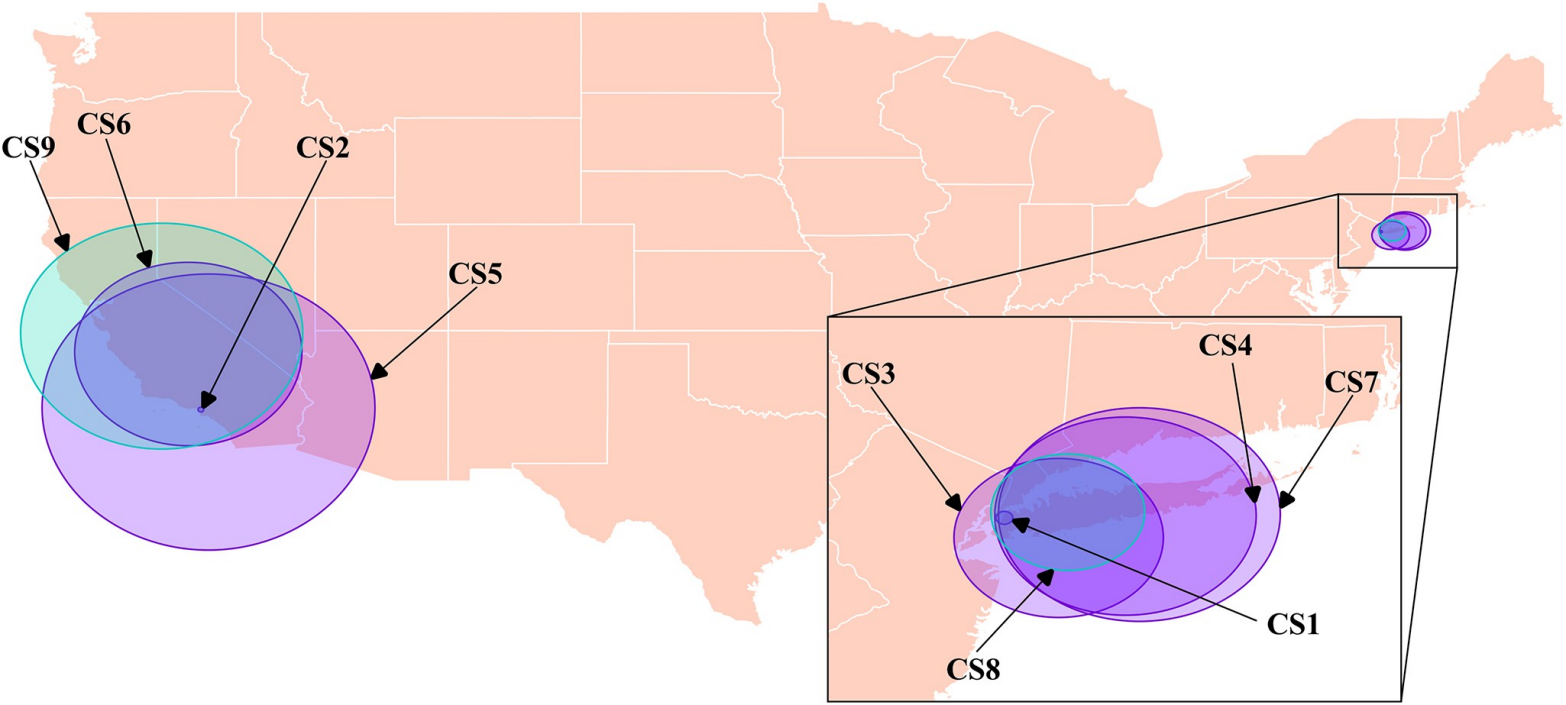
Locations of statistically significant spatial clusters identified with the spatial scan statistic using Bernoulli models, where there were statistically significant higher proportions of dog-associated poisoning calls to the Animal Poison Control Center related to cannabinoids in the continental US (2005–2014).

The purely spatial scans identified 9 statistically significant spatial clusters of proportionally high cannabinoid calls (Fig 5 & Table 6). These clusters generally centered around, southern California and Long Island, New York. We identified cluster CS1 (Cannabinoid Spatial 1) in the 2005 scan, which covered the west side of Long Island and the southern half of Manhattan, in New York City, and was centered just south of Long Island City (Fig 5 & Table 6). Another small cluster, CS2, was identified in the 2007 scan, and covered the western half of Los Angeles, California, and was centered over the West Adams area of Los Angeles (Fig 5 & Table 6). CS3, a 2008 cluster, CS4 a 2010 cluster, and CS7 a 2014 cluster, all covered a similar region which included the majority of Long Island, with CS4 and CS7 including Staten Island and part of northeastern New Jersey (Fig 5 & Table 6). Clusters CS5 and CS6 identified by the 2012 and 2014 scan tests, respectively, both covered the majority of the southern half of California and Nevada, but cluster CS5 also included western Arizona (Fig 5 & Table 6). When the scan test was performed on the entire study period, CS8 and CS9 were identified, and they covered the western half of Long Island and most of California and Nevada, respectively (Fig 5 & Table 6).

**Table 6:** Purely spatial clusters identified with the spatial scan statistic using Bernoulli models, where there were statistically significant higher proportions of dog-associated poisoning calls to the APCC^a^ related to cannabinoids in the continental US (2005–2014).

Cluster	Year(s) Scanned	O/E^b^	Relative Risk	Observed	Expected	P-Value	Latitude	Longitude	Radius (Km)
CS1	2005	**9.51**	**10.24**	8	0.84	0.032	40.73	-73.95	4.82
CS2	2007	**9.71**	**10.28**	11	1.13	0.001	34.03	-118.35	9.96
CS3	2008	**3.19**	**3.82**	38	11.91	0.001	40.59	-73.58	59.44
CS4	2010	**2.65**	**3.03**	43	16.24	0.001	40.74	-73.13	74.06
CS5	2012	**1.95**	**2.26**	53	27.20	0.044	33.94	-118.07	577.12
CS6	2014	**1.96**	**2.25**	82	41.74	0.001	36.12	-118.83	383.61
CS7	2014	**2.32**	**2.53**	49	21.10	0.004	40.75	-73.04	79.84
CS8	2005–2014	**2.17**	**2.32**	230	106.03	<0.001	40.76	-73.52	43.60
CS9	2005–2014	**1.60**	**1.75**	405	253.28	<0.001	36.79	-119.83	472.16

^a^Animal Poison Control Center

^b^Observed/Expected

#### Purely temporal clusters

When the temporal scan test was performed for the entire study period, it identified a single statistically significant temporal cluster (CT1) of proportionally high cannabinoid calls from June 29, 2010 to December 28, 2014 (Table 4). We did not identify any temporal clusters when the scan was performed on each year individually.

#### Space-Time clusters

**Fig 6:**
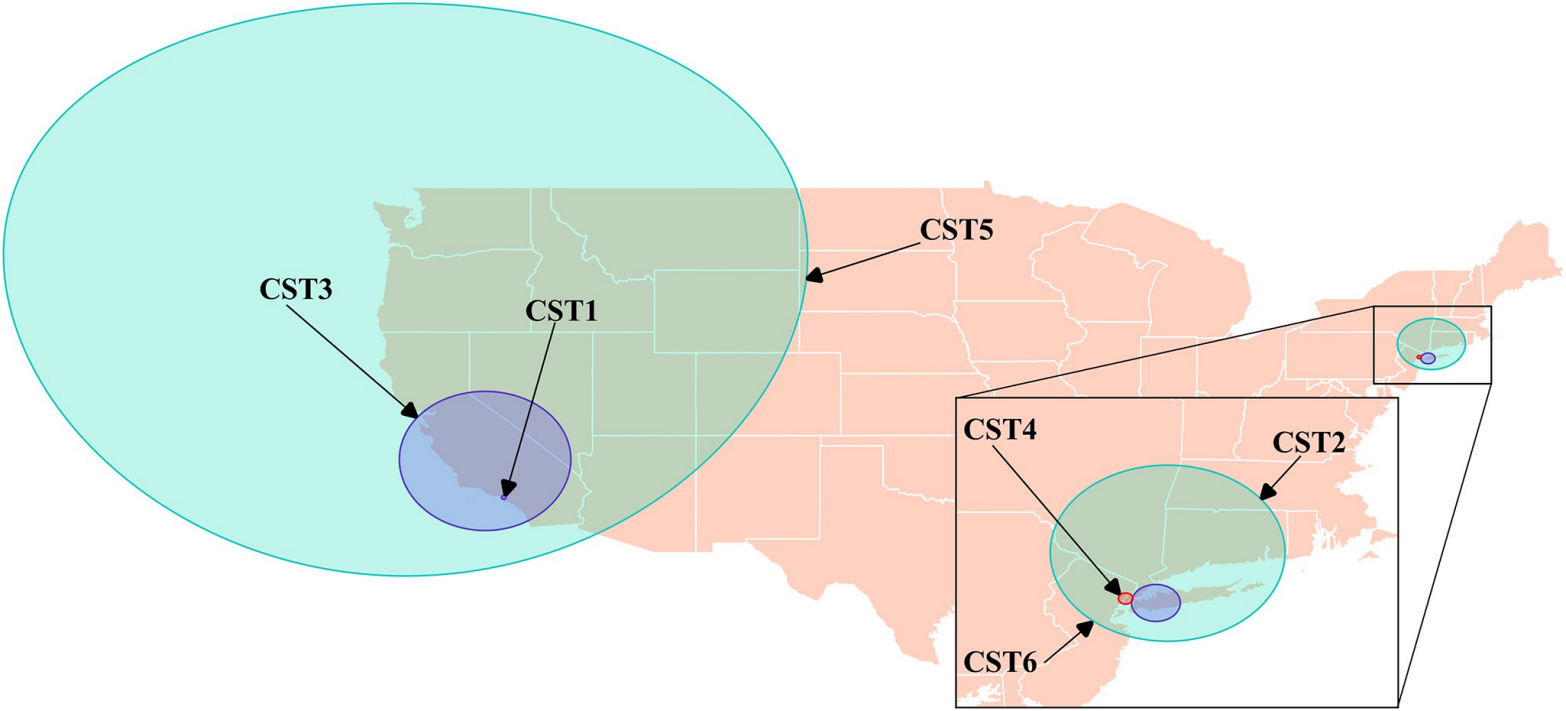
Locations of statistically significant space-time clusters of high levels of calls concerning cannabinoids identified using the spatial scan statistic with Bernoulli and space-time permutation models, based on data from dog-associated poisoning calls to the Animal Poison Control Center related to cannabinoids in the continental US (2005–2014).

Using scan tests with yearly data, we identified statistically significant space-time clusters using Bernoulli (CST1, CST2, CST3) and space-time permutation (CST4) models (Fig 6 & Table 7). Like the purely spatial analyses, these clusters centered around/near southern California or New York City. We identified CST1 (Cannabinoid Space-Time 1), occurring from April 1 to June 30, 2007, covering eastern Los Angeles, and was centered over the Crenshaw area of the city (Fig 6 & Table 7). Cluster CST2 occurred from May 1 to October 31, 2008, covered the western half of Long Island, and was centered over Garden City, New York (Fig 6 & Table 7). Cluster CST3 covered most of southern California, was centered over Delano from July 1 to December 31, 2014 (Fig 6 & Table 7). Cluster CST4 covered northern Jersey City and central Manhattan and occurred from June 1 to June 30, 2014 (Fig 6 & Table 7). When the scan statistic was performed on the entire study period using the space-time Bernoulli model, CST5 and CST6 were identified, and they covered the entire west coast from January 1, 2010 to December 31, 2014 and eastern New York state from January 1, 2010 to December 31, 2014, respectively (Fig 6 & Table 7). No clusters were identified when the space-time permutation model was applied to the entire study period.

**Table 7:** Space-time clusters identified with the spatial scan statistic using Bernoulli and space-time permutation models, where there were statistically significant higher levels of dog-associated poisoning calls to the APCC^a^ related to cannabinoids in the continental US (2005–2014).

Cluster	Model Type	Year(s) Scanned	Start Date	End Date	O/E^b^	Relative Risk	Observed	Expected	P-Value	Latitude	Longitude	Radius (Km)
CST1	Space-Time Bernoulli	2007	4/1/2007	6/30/2007	**30.12**	**31.37**	7	0.23	0.003	34.01	-118.34	10.64
CST2	Space-Time Bernoulli	2008	5/1/2008	10/31/2008	**6.83**	**7.39**	15	2.20	0.004	40.73	-73.64	28.78
CST3	Space-Time Bernoulli	2014	7/1/2014	12/31/2014	**2.76**	**3.07**	55	19.95	0.001	35.77	-119.25	373.86
CST4	Space-Time Permutation	2013	6/1/2013	6/30/2013	**12.74**	**N/A**	5	0.39	0.031	40.79	-74.06	8.57
CST5	Space-Time Bernoulli	2005–2014	1/1/2010	12/31/2014	**1.75**	**1.93**	380	216.67	0.001	44.05	-123.09	1538.44
CST6	Space-Time Bernoulli	2005–2014	1/1/2010	12/31/2014	**1.85**	**1.99**	291	157.20	0.001	41.42	-73.47	137.01

^a^Animal Poison Control Center

^b^Observed/Expected

## Discussion

This study provides the first US national population-based analysis identifying spatial and/or temporal clusters associated with opioid and cannabinoid poisonings in dogs. Using call data provided by the ASPCA concerning dog poisonings reported to the APCC, we created the first state-level choropleth maps depicting the distribution of opioid and cannabinoid poisoning events in dogs. We applied spatial scan statistics to identify clusters within mainland US in space, time, and space-time, that show where and when there were higher proportions/levels of calls that reported either opioid or cannabinoid poisoning events in dogs.

### Opioids analyses

The choropleth map depicting the proportional distribution of opioid poisoning calls shows which regions dogs were at the highest risk of opioid poisonings at the state level (Fig 1 and Table 2). The map highlights that dog opioid poisonings were highest in West Virginia, Oklahoma, Utah, Louisiana, Michigan, Arkansas, Nevada, and Delaware. These states group in four regions, the northern Appalachian area around Western Virginia to Michigan, the areas around Arkansas, Oklahoma, and Louisiana, and the areas around Nevada and Utah, and Delaware. The depiction of the distribution of opioid dog poisonings in the choropleth map is for the most part consistent with the spatial and space-time clusters identified in this study. The opioid choropleth map in this study shows a pattern similar to the choropleth maps depicting human opioid prescription rates [29], with states like West Virginia, Louisiana, Arkansas, Oklahoma having both very high proportional dog opioid poisoning events and prescription rates. These findings are consistent with our previous work, which also showed a positive association between opioid prescription rates and dog opioid poisoning events [12]. However, there are clear exceptions to this, where states like Mississippi and Tennessee had very high prescription rates but a relatively low proportion of calls related to opioid dog poisonings. A similar scenario exists with state-level human opioid death rates [30], where states like West Virginia and Utah had a high proportion of calls related to opioid dog poisonings and high human opioid-related death rates. However, there are also many major exceptions, for example, Arkansas and Louisiana had a high proportion of calls related to opioid dog poisonings and high prescription rates but had very low human opioid-related death rates. These differences may reflect the nature of the opioids being used within these regions in terms of whether they are acquired through prescriptions or illicitly and whether they are adulterated with more potent opioids (e.g., fentanyl and carfentanil) [12, 29].

The space, time, and space-time analyses detected several clusters identifying when and where there were particularly high opioid poisonings in dogs in the US (Figs 3 and 4 & Tables 3–5). The purely temporal analyses identified a single cluster in the first half of the study when the analysis was performed over the entire study period (Table 4). This coincides with the peak years of opioid poisoning events in dogs from this dataset [12]. No evidence of a seasonal pattern was detected. This is important because there is evidence to suggest cannabis and opioid use vary substantially by season [31, 32], and that dog behaviours which may be related to exposure, such as time spent outdoors, may also vary by season. Opioid poisonings increased from 2005 to 2008 where it peaked, then decreased to its lowest in 2014. Spatial and spatiotemporal clusters identifying where there were higher levels of opioid dog poisoning calls tended to appear in one of three regions and all occurred before 2011 and were fairly consistent with human use data, as seen in Appalachian areas [21, 22].

County-level human opioid death rates show that the northern Appalachian area, surrounding West Virginia, Kentucky, Ohio, and western Pennsylvania, was the area where humans deaths were proportionally the highest [21]. County-level human opioid prescription rates show that prescription opioids were most prevalent in the mideastern US, stretching from this northern Appalachian area to the northern Gulf of Mexico region [22]. Although not completely overlapping, similarities in the results reported from human data and the clusters identified using dog data were noted. Clusters OS1, OS3, and OST3 covered this region in the mideastern US (Figs 3 and 4 & Tables 3–5). Cluster OS1 in 2005 encompassed the northern Appalachian area of Ohio, West Virginia, and Pennsylvania. Cluster OS3 in 2010 covered the same area but included more of the Appalachian area and the Delaware Valley (Fig 3 & Table 3). From 2005 to 2010 OST3 encompassed the entire mideastern US, including the same area as OS3 and additional areas around Arkansas and Louisiana, and covered the same period as the purely temporal clusters we identified (Figs 3 and 4 & Tables 3–5). These clusters covered the US regions that have both, the highest human opioid prescription rates, and death rates [21, 22].

A second group of opioid spatial and spatiotemporal clusters overlapped in space: OS2, OS5, OST1, and OST2 (Figs 3 and 4 & Tables 3 and 5). These clusters occurred in the Delaware valley and its surrounding areas where Delaware state, Pennsylvania, Maryland, and New Jersey meet, with OST1 covering most of the Delaware county of west Philadelphia. These clusters occurred in a region where heroin use was known to be prevalent [33], and included some counties with high death and/or prescription rates associated with opioids [21, 29, 30].

The spatial cluster based on a scan of all data from 2005–2014 was very large in diameter and covered most of the country (Fig 3 & Table 3). It captured areas identified by the annual scan analyses, but also included some western states with notably high levels of opioid calls over the entire study period (Fig 1).

It is apparent that dog opioid poisoning events were not evenly distributed in space or time. Further studies are needed to understand why dog poisoning events occurred more often in some regions than others. However, human opioid prescription rates, death rates associated with opioids, and heroin use seem to indirectly measure the level of opioids in a dog’s environment.

### Cannabis analyses

The choropleth map depicting the state-level proportion of cannabinoid poisoning calls gives a general view of which regions dogs were at the highest risk of a cannabinoid poisoning event (Fig 2 & Table 2). At the state-level, the proportion of calls related to cannabinoid poisonings were highest in Montana, New York, District of Columbia, California, Washington, Massachusetts, Nevada, and New Mexico. Choropleth maps depicting human cannabinoid use typically indicated Washington, Oregon, Colorado, District of Columbia, and Massachusetts as being regions of prevalent cannabinoid use but also included California, New York, New Mexico, and Montana as moderately high [24]. However, the maps also depict Maine, Vermont, New Hampshire, and Rhode Island as high, which had middle to low levels of cannabinoid related calls about dog poisoning events.

Based on current data, the highest prevalence of human cannabinoid use tends to be centered in four regions, the areas around the far northwest, the far northeast, Colorado, and the District of Columbia [24]. However, our analyses highlighted two regions where the proportion of calls related to cannabinoid poisoning events were particularly high. All clusters identified encompassed Los Angeles and large portions of California, as well as New York City and the areas around Long Island (Figs 4 and 5 & Tables 5 and 6). The purely temporal analyses identified a single cluster in the second half of the study (2010–2014) when the analysis was performed on the entire study period (Table 4). This temporal cluster coincides with the peak years of cannabinoid poisonings in dogs from this dataset [12]. Like opioids, no evidence of a seasonal trend was found. Similar to previous work, cannabinoid poisoning events increased throughout the period of this study [13, 15].

Clusters CS1, CS3, CS4, CS7, CS8, CST2, CST4, and CST6 encompassed areas of New York City and neighbouring counties in New Jersey (Figs 4 and 5 & Tables 5 and 6). These clusters were consistent with trends identified in our choropleth map. Although the only purely temporal cluster occurred in the second half of the study, these spatial and space-time clusters for the New York City/Long Island region occurred throughout the study period. Interestingly, at the substate-level, the New York City/Long Island region had only moderate human cannabinoid use [23].

Clusters CS5, CS6, CS9, and CST3 encompassed counties around southern California, and clusters CS2 and CST1 encompassed areas of East and West Los Angeles, respectively, while CST5 encompassed the entire West Coast of the US (Figs 4 and 5 & Tables 5 and 6). The clusters identified were consistent with trends identified in our choropleth map (Fig 2). Human cannabinoid use has been noted to be particularly high in the northern half of the West Coast, stretching from northern California to northern Washington [23, 24]; this region was captured by the space-time cluster CST5, which occurred form 2010–2014. Within California, human cannabinoid use trends also highlighted a region roughly from San Francisco south to San Luis Obispo, a region captured by clusters CS5, CS6, CS9, and CST3 [23]. CS2 and CST1 were consistent with Los Angeles being a moderately high-risk region for cannabinoid poisonings in dogs.

Our recent work suggests there is an association between cannabis legislation and cannabis poisonings in dogs [13]. The results from this study are consistent with this theory. In this study, the majority of spatial and space-time clusters occurred in regions where and when cannabis use and possession was decriminalized, with only CS1 and CST2 centered in a region (i.e., New York state) where use and possession were restricted at the time. The temporal cannabinoid poisoning cluster occurred in the latter half of the study period when more regions had reduced penalties for cannabis use and possession. Given the trend of cannabis legalization throughout the study period, it should be noted that the increase in cannabinoid poisoning calls may be a result of a combination of increased human use and reduced fears in reporting cannabinoid poisonings concerning a substance that was previously illegal [13].

## Conclusion

As human opioid use and cannabinoid use and acceptance continues to increase, it is of growing importance to understand how these changes will affect different populations. By identifying when and where dogs were at the greatest risk of opioid or cannabinoid poisonings, governing bodies, public health departments, and veterinarians may use this information to help guide future studies in these regions and direct possible interventions. This study highlights that the risk of calls related to opioid and cannabinoid poisoning events was clustered in space, time, and space-time. While the use of self-reported call center data may result in biases, it was noteworthy that the patterns of calls concerning dogs were frequently consistent with human patterns. In addition, the patterns of clusters were distinct between opioid and cannabinoid calls, highlighting that a bias in calls related to the social stigma around drugs could not explain our results. The risk of a dog opioid poisoning seems to have reduced throughout the study while the risk of a cannabinoid poisoning seemed to have increased. Although there is evidence that seasonal trends exist for human opioid and cannabinoid use, this study identified no seasonal trends for either opioid or cannabinoid poisonings in dogs. With potentially problematic regions identified for dog poisonings with opioids and cannabinoids, we hope this study helps direct future research to identify factors that influence risk and possibly guide interventions aimed at educating the public about safeguarding their drugs to prevent vulnerable populations, including children and pets, from accidental poisonings.
